# A Network Model for Detecting Marine Floating Weak Targets Based on Multimodal Data Fusion of Radar Echoes

**DOI:** 10.3390/s22239163

**Published:** 2022-11-25

**Authors:** Guoxing Duan, Yunhua Wang, Yanmin Zhang, Shuya Wu, Letian Lv

**Affiliations:** 1 Faculty of Information Science and Engineering, Ocean University of China, Qingdao 266100, China; 2 Pilot National Laboratory for Marine Science and Technology, Qingdao 266237, China

**Keywords:** radar target detection, marine floating weak targets, multimodal data fusion, deep learning

## Abstract

Due to the interaction between floating weak targets and sea clutter in complex marine environments, it is necessary to distinguish targets and sea clutter from different dimensions by designing universal deep learning models. Therefore, in this paper, we introduce the concept of multimodal data fusion from the field of artificial intelligence (AI) to the marine target detection task. Using deep learning methods, a target detection network model based on the multimodal data fusion of radar echoes is proposed. In the paper, according to the characteristics of different modalities data, the temporal LeNet (T-LeNet) network module and time-frequency feature extraction network module are constructed to extract the time domain features, frequency domain features, and time-frequency features from radar sea surface echo signals. To avoid the impact of redundant features between different modalities data on detection performance, a Self-Attention mechanism is introduced to fuse and optimize the features of different dimensions. The experimental results based on the publicly available IPIX radar and CSIR datasets show that the multimodal data fusion of radar echoes can effectively improve the detection performance of marine floating weak targets. The proposed model has a target detection probability of 0.97 when the false alarm probability is $10^{-3}$ under the lower signal-to-clutter ratio (SCR) sea state. Compared with the feature-based detector and the detection model based on single-modality data, the new model proposed by us has stronger detection performance and universality under various marine detection environments. Moreover, the transfer learning method is used to train the new model in this paper, which effectively reduces the model training time. This provides the possibility of applying deep learning methods to real-time target detection at sea.

## 1. Introduction

In the complex and changing maritime observation environment, radar is used as one of the main tools for marine target detection. How to use radar echoes for the detection of marine targets has been challenging [1,2,3,4]. With the miniaturization and invisibility of marine targets, for small floating targets such as floating boats, buoys, and frogmen, the radar cross section (RCS) of these targets is small and the echo signal is weak, which makes the signal-to-clutter ratio (SCR) of target and sea clutter low. Moreover, the sea clutter is non-uniform, non-smooth, and non-Gaussian over temporal and spatial variations [5,6,7,8], resulting in the targets’ RCS and SCR being seriously influenced. In the Doppler domain, due to the relatively slow motion of the floating target on the sea surface, it is extremely easy to be obscured or submerged by the sea clutter [9,10].

For the interference of sea clutter such as sea spikes and white waves, the target detection algorithms based on statistical theory [11,12,13,14] are difficult to satisfy the demand of target detection under different sea states. Some researchers have proposed target detection algorithms based on different echo features, which can alleviate the occurrence of false alarm events and missed alarm events during target detection to some extent. In 1993, T. Lo et al. introduced nonlinear fractal theory [15] based on the fluctuation analysis of sea surface and proposed detectors based on features such as the normalized Hurst exponent [16] and fractal dimension [17], respectively. However, in scanning mode radar observation, the performance of the detector based on fractal theory degrades due to the relatively short observation time of the target. To improve the target detection performance, Shui et al. proposed a Tri-feature-based detector [18] by using three features, relative amplitude, relative Doppler peak height and relative entropy, extracted from the amplitude sequence and Doppler amplitude spectrum of the echo signal, respectively. However, in the Doppler domain, it is difficult to distinguish the target and sea clutter based on the features when the target echo falls into the sea clutter region. In the short-time echo accumulation, the target echo and sea clutter exhibit different time-frequency (TF) characteristics. Shui et al. further extracted three TF features of ridge integration, the number of connected regions, and the maximum size of connected regions using the time-Doppler spectrum image. Then, a tri-feature-based detector using the TF features [19] was constructed, which improved the target detection performance.

Currently, there are also detectors based on multi-feature fusion such as the K-Nearest Neighbor algorithm [20] and adaptive threshold detection [21]. However, the knowledge of target characteristics and sea clutter characteristics is still in the exploration stage [22]. It is difficult to obtain universal statistical features to accurately distinguish the targets and sea clutter. So that the feature-based detection methods are limited in improving the target detection performance. In recent years, with the emergence of Convolutional Neural Networks (CNN), Recurrent Neural Networks (RNN), and other deep learning network models, some researchers have applied them to the field of marine target detection [23]. In [24], a VGG network model was used to classify time-Doppler spectrum images of different marine simulation targets. However, for the measured sea surface echo data, the sea clutter sometimes exhibits similar features to moving targets in the time-Doppler spectrum image, which leads to classification errors. In [25], the RNN network model was used to extract the temporal features of the amplitude sequence in the sea surface echoes. Due to the Doppler information of the echoes is not utilized, there is a high error rate by only using the amplitude information. To design high-performance detection network models, the Graph Convolutional Network (GCN) model and the Faster R-CNN network model were used in [26,27] for marine target detection tasks. Because the radar detection environment involves different target characteristic, it is unreliable to use a single datum. To utilize the amplitude sequences and time-Doppler spectrum images of sea surface echoes, a two-channel CNN network model was constructed in [28] to classify targets and sea clutter. However, the detection performance of the target is degraded due to the redundant features between the data not being considered. The results from various scholars show that the deep learning network model has strong feature extraction capability and good generalization ability. It can be better applied to marine target detection tasks.

For marine floating weak targets in the complex sea clutter background, it is difficult to achieve the high-performance detection of targets due to the interaction between sea clutter and targets. To solve this problem, it is necessary to obtain echo data with different properties to characterize the target and sea clutter from different perspectives. In addition, the design of the universal detection network models based on the characteristics of echo datum is one of the effective ways to achieve high-performance marine target detection. In the field of Artificial Intelligence (AI), multimodal data can characterize different forms of the same thing. The multimodal data such as images, speech, and text can be obtained by multiple sensors. The perception of things by deep learning network models can be enhanced using multimodal data fusion [29,30]. However, characterizing the marine target by multiple radar sensors is difficult in radar marine target detection tasks. So, generally, a single radar sensor is used to observe the marine target. To achieve high performance detection of marine floating weak targets by using a single radar, its echo characteristics need to be fully explored and utilized. In this paper, the sea surface echo signals from a single radar are processed in the time domain, frequency domain, and time-frequency domain to obtain three modalities data: the amplitude sequence, the Doppler amplitude spectrum, and the time-Doppler spectrum image. The data of these three modalities can characterize the temporal and spatial variations of the target and sea clutter from different perspectives.

Therefore, for mining the characterization capability of different modality data, in this paper, we introduce the concept of multimodal data fusion into the radar marine target detection task. Using deep learning techniques, a target detection network model based on the multimodal data fusion of radar echoes is proposed according to the characteristics of different modalities data. The temporal LeNet (T-LeNet) network module is designed based on the serial correlation property of the amplitude sequence and the Doppler amplitude spectrum. The ResNet50 network model [31] is used to obtain a time-frequency feature extraction network module for the time-Doppler spectrum image processing. In addition, to avoid the redundant features between different data, a Self-Attention mechanism [32] is introduced in this paper for fusing as well as optimizing the different echo features. Finally, the target and sea clutter are discriminated by decision thresholds with different false alarm probabilities ($P_{fa}$). In the model training process, we utilize the transfer learning method to initialize the model parameters, which greatly reduces the model training time. Compared with the deep learning detection methods and feature-based detection methods in the literature, the proposed new model in this paper utilizes the data of different modalities in radar sea surface echoes and achieves better detection results.

To demonstrate the effectiveness of the proposed model in this paper, we conduct target detection experiments under different SCR sea state conditions based on the publicly available IPIX radar dataset [33]. From the comparison of detection results, it can be concluded that the proposed model has better detection results and stronger stability than both the feature-based detection method [18,19] and the detection method based on single-modality data under lower SCR sea states. When the false alarm probability is $10^{-3}$, the detection probability of the proposed model is 0.97. The detection probabilities of the Tri-feature-based detector [18] and the Feature-based detector using three TF features [19] are 0.04 and 0.79, respectively, and the detection probabilities of the single-modality data based on the amplitude sequence, the Doppler amplitude spectrum, and the time-Doppler spectrum image are 0.45, 0.05, and 0.33, respectively. In addition, experiments on different marine floating targets based on the publicly available CSIR dataset [34] show that the proposed model also has strong universality compared with the feature-based detector under different detection conditions.

The paper is organized as follows. Section 2 provides a brief description of the IPIX dataset and CSIR dataset and the multimodal data are analyzed. In Section 3, the characteristics of different modalities data are used to construct a target detection network model. Additionally, in Section 4, the experimental validation and results analysis are based on the IPIX radar dataset and CSIR dataset. Finally, in Section 5, we conclude our paper.

## 2. Dataset Introduction and Multimodal Data Analysis

### 2.1. Dataset Introduction

In this section, we briefly describe the basic information of the radar data used in this paper. The data #IPIX_01, #IPIX_02, and #IPIX_03 in Table 1 were obtained by the IPIX radar under three different sea state conditions [33]. The IPIX radar works in the staring mode with a radar carrier frequency of 9.36 GHz. The pulse repetition frequency (PRF) of the radar data in Table 1 is 1000 Hz. All the data have an observation time of 131.072 s. The data consist of 14 range cells and the range resolution is 30 m. The radar observation target is an anchored spherical block of Styrofoam wrapped with wire mesh and its diameter is about 1 m. During the radar observation time, the target floats on the sea surface, rising and falling with the waves. As shown in Table 1, we define the target location as the target cell, the region affected by the target as the guard cells, and the other regions as the sea clutter cells.

(1) $$\mathrm{SCR}=10\log_{10}(\frac{{\bar{p}}_{t}-{\bar{p}}_{c}}{{\bar{p}}_{c}})$$

In addition, the IPIX radar was mounted on a cliff of 30 m height on the east coast of Canada. Due to the local gusts, the wind speed (WS) was variable and the observed sea surface was affected by breaking waves and whitecaps, which makes the power of the sea clutter enhanced. Table 1 lists the relevant parameters of different IPIX radar data, including the WS, significant wave height (SWH), the angle between the line of radar sight and the wind direction, and the SCR of different data. In this paper, Equation (1) is used to estimate the SCR of radar echo data, in which ${\bar{p}}_{c}$ is the average power of the echo signal in all the sea clutter cells and ${\bar{p}}_{t}$ is the average power of the echo signal in the target cell. By calculation, we can obtain SCR results that are almost consistent with those in [18,19].

Since the measured SWH is the result of the combined effect of wind waves and swells, #IPIX_03 and #IPIX_02 in Table 1 have the same WS, while the SWH of #IPIX_03 is higher, so we can roughly infer that the SWH of #IPIX_03 is mainly influenced by the swell. Additionally, the waves of #IPIX_03 may cause the obscuring effect on part of the observed region because of the low grazing angle (about 0.33°) of the radar observation and the higher SWH of the sea surface. Therefore, the calculated that the average power of the sea clutter is relatively low, while the SCR of #IPIX_03 estimated by Equation (1) is larger. For #IPIX_01 and #IPIX_02, they were obtained by radar crosswind observations of the targets. Because the WS of #IPIX_01 is larger, which causes the greater change of the sea surface motion state, the energy of the sea surface echo is enhanced, while the energy of the target echo is relatively weaker. Therefore, the SCR of #IPIX_01 calculated by Equation (1) is smaller. The results of the above analysis show that the SCR variation of the radar echo data is determined by many factors such as SWH, wind direction, WS, radar observation geometry, etc., and it does not have to be strictly monotonic increasing or decreasing with the sea state.

The multiple CSIR data [34] are shown in Table 2. The observed target for data #CSIR_01 and #CSIR_02 is a floating fishing boat, and the observed target for data #CSIR_03 and #CSIR_04 is a floating Rigid Inflatable Boat (RIB). The PRF of the CSIR data in Table 2 is 5000 Hz and there is some variability in the radar observation time as well as the SCR values of the echo data. The radar data consist of 48 or 96 range cells and the range resolution is 15 m. The radar works in a staring mode. The carrier frequency of #CSIR_01 and #CSIR_02 is 6.9 GHz and the carrier frequency of #CSIR_03 and #CSIR_04 is 9 GHz. During the radar observation time, the floating fishing boat and the floating RIB remain in the same range cell. Comparing the data of #CSIR_03 and #CSIR_04, it can be seen that the SCR values have some differences under the same SWH environment due to the influence of observation geometry, WS, and other factors.

**Figure 1:**
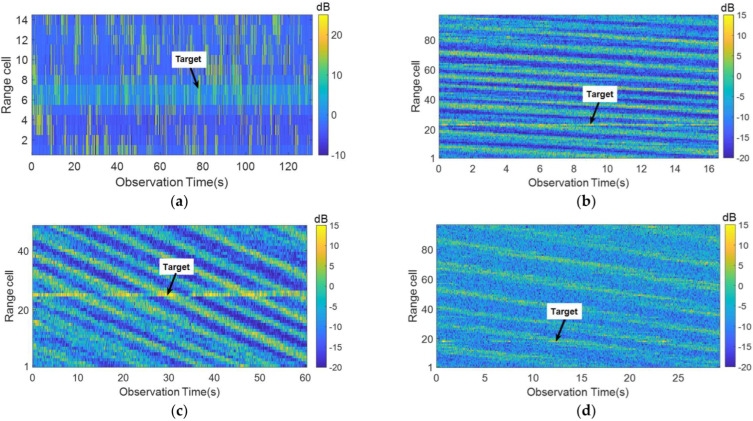
Radar echo intensity images of different observed targets. (**a**–**d**) show the intensity images of data #IPIX_01, #CSIR_01, #CSIR_02, and #CSIR_03, respectively. The arrow points to the target motion trajectory. (**a**) The floating ball; (**b**) The floating fishing boat; (**c**) The floating fishing boat; (**d**) The floating RIB.

Figure 1 shows the radar echo intensity images of different observation targets. Within the radar observation time of tens or even hundreds of seconds, we can clearly see the direction of the wave motion and the change of the radar echo intensity. Because the echo energy of sea spikes, white waves, and other sea clutter are strong, it is more difficult to accurately identify the target by comparing the change in echo energy. Therefore, it is necessary to combine other signal processing methods to improve the detection ability of marine floating weak targets.

### 2.2. Multimodal Data Analysis of Radar Echoes

In this section, we obtain three types of modalities data: the amplitude sequence, the Doppler amplitude spectrum, and the time-Doppler spectrum image by processing the radar echo signals in the time domain, frequency domain, and time-frequency domain, respectively. The situation of the target and sea clutter is characterized from different perspectives.

**Figure 2:**
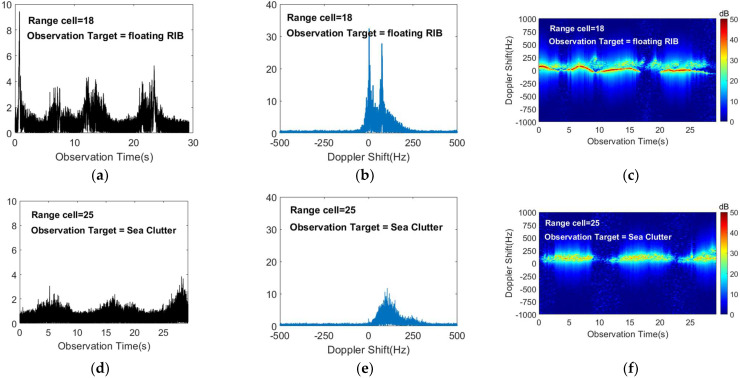
The multimodal data of radar echo signals in the target cells and the sea clutter cells. The radar observation target is a floating RIB and the radar echo signal sequence is the 18th and 25th range cells of the data #CSIR_03. (**a**) Amplitude sequence of the target cell; (**b**) Doppler amplitude spectrum of the target cell; (**c**) time-Doppler spectrum image of the target cell; (**d**) Amplitude sequence of the sea clutter cell; (**e**) Doppler amplitude spectrum of the sea clutter cell; (**f**) time-Doppler spectrum image of the sea clutter cell.

We obtain the amplitude sequence by calculating the modulus for the long-time radar echo signal sequence x=[x(1),x(2),…,x(n)]. The amplitude sequence variations of the target cell and the sea clutter cell in the data #CSIR_03 are shown in Figure 2a,d, respectively. It can be seen that the amplitude of the target is stronger than that of the sea clutter. The amplitude variation of the target cell also shows periodicity because the floating target is affected by the periodic motion of the waves.

The Doppler amplitude spectrum of the target cell and the sea clutter cell in the data #CSIR_03 are shown in Figure 2b,e, respectively, and the radar observation target is a floating RIB. We use Fourier transform theory to obtain the Doppler amplitude spectrum by processing the echo signal sequence according to Equation (2). In the spectrum, the motion of the target and the sea clutter can be described according to the energy distribution on different frequency bins.

(2) $$S(f_{d})=\frac{1}{\sqrt{N}}\left|\sum_{n=1}^{N}x(n)\exp(-2\pi f_{d}nT_{r})\right|,f_{d}\in [-\frac{1}{2T_{r}},\frac{1}{2T_{r}}]$$

where $f_{d}$ is the Doppler frequency, $T_{r}$ is the pulse repetition interval (PRI) of the radar, and N is the length of the echo signal sequence.

By comparing the Doppler amplitude spectrum of the two range cells, it can be concluded that the motion of the target is mainly concentrated in zero-frequency during the long-time coherent accumulation, while the motion of the sea clutter is mainly concentrated around 100 Hz. The target and sea clutter can be distinguished to some extent according to the difference of Doppler shift. It can also be seen from Figure 2b that due to the similarity between the motion state of the floating target and the sea surface, the target echo will fall into the sea clutter region and it is difficult to identify the target in the short-time coherent accumulation. However, in the long-time coherent accumulation, the target echo energy is stronger and the target can be identified in the sea clutter region. In addition, because the Doppler shift of the sea clutter motion is positive, it can be roughly estimated that the waves are moving toward the radar position.

In the short-time coherent accumulation, because of the target and the sea clutter exhibit different TF characteristics, we use the smoothed Pseudo-Wigner-Ville distributions (SPWVD) [19,35] in Equation (3) to process the echo signal sequence to obtain the time-Doppler spectrum image. The spectrum image can describe the motion of the target and the sea clutter, which vary with the time of radar observation.

(3) $$\mathrm{SPWVD}(n,l|x)=\sum_{m=-M}^{M}g(m)\sum_{k=-K}^{K}h(k)x(n+m+k)x^{*}(n+m-k)\exp(-j4\pi kl\Delta f_{d})$$

where the superscript “*” denotes the conjugate, $\Delta f_{d}$ is the sampling interval of the normalized Doppler frequency. It is a separable TF smoothed version of the discrete Wigner–Ville distribution, where g(m) and h(k) are the time and frequency smoothing windows, respectively, M and K are the sizes of the time and frequency smoothing windows, respectively.

The time-Doppler spectrum of the target cell and the sea clutter cell in the data #CSIR_03 are indicated in Figure 2c,f, respectively. It is obvious that the target makes a periodic motion around zero-frequency, forming a relatively continuous motion trajectory. Since the spectral images of the target and sea clutter cells exhibit large differences in the spatial distribution of energy, the use of this property is beneficial for target and sea clutter discrimination [19].

## 3. Construction and Training of Target Detection Network Model

### 3.1. Construction of Network Model

**Figure 3:**
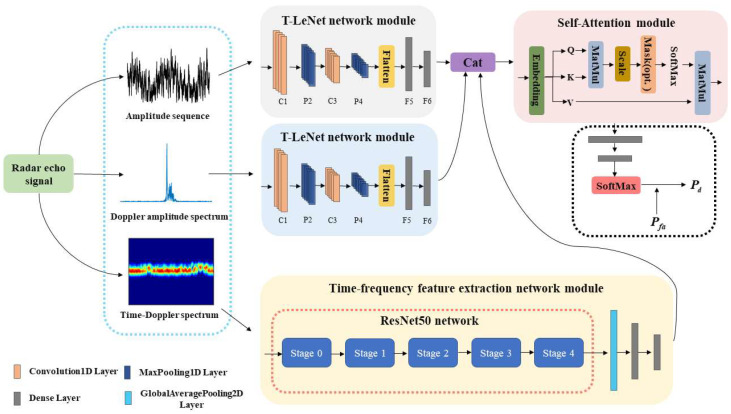
The flowchart of the proposed target detection network model.

In different sea states, it is difficult to distinguish targets by only using the time-Doppler spectrum and the Doppler amplitude spectrum image due to the similarity of frequency characteristics of the floating targets and sea clutter. The target detection methods that only use amplitude information also have high error rates because the Doppler information of the echoes is not utilized. When the target falls into the region of strong sea clutter, the Doppler amplitude spectrum can compensate the problem that the time-Doppler spectrum image cannot distinguish the target and sea clutter, so we enhance the detection ability of the target by using the Doppler amplitude spectrum in the frequency domain. Combining various characteristics of the echo data can describe the target and sea clutter from different perspectives. It is one of the effective ways to achieve high-performance detection of marine floating weak targets. Therefore, using deep learning techniques, a target detection network model based on the multimodal data fusion is proposed by studying the different characteristics of amplitude sequences and Doppler amplitude spectra and time-Doppler spectrum images. The structure of the target detection network model is shown in Figure 3.

**Figure 4:**
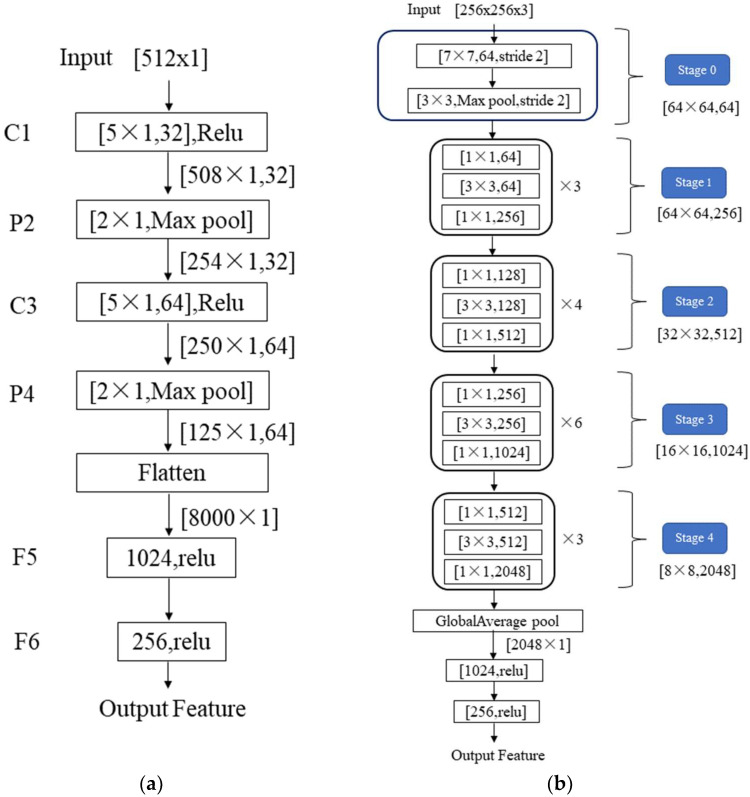
T-LeNet network module (**a**) and time-frequency feature extraction network module (**b**). In (**a**), it contains seven network layers. In (**b**), from Stage1 to Stage4, each module contains a Conv Block (located in the first block) and multiple Identity Blocks.

This network model includes feature extraction, feature fusion, and target detection. In the feature extraction stage, the features of different modalities data are obtained through the T-LeNet network module and the time-frequency feature extraction network module. The motion state of the floating target and the sea surface is slowly changing with time, which makes the energy distribution in the amplitude sequence and the Doppler amplitude spectrum image have a strong serial correlation. Therefore, in this paper, the features of the amplitude sequence and the Doppler amplitude spectrum are extracted by the T-LeNet network module in Figure 4a. In Figure 4a, C1 and C3 are 1D convolutional network layers, P2 and P4 are 1D Max pooling network layers, and F5 and F6 are fully connected network layers. The rectangular boxes contain the parameters of the network layer, such as the number of neurons, the convolutional kernel scale and number, the activation function (Relu), and the pooling mode (Max Pooling). The output feature dimension of the network layer is shown below each rectangular box. We obtain the T-LeNet network that can handle time series data by optimizing the LeNet network model [36]. The LeNet network model is a very efficient convolutional neural network for handwritten character recognition and the network contains a total of two 2D convolutional layers, two 2D Max pooling layers, and three fully connected layers. According to the temporal characteristics of the amplitude sequence and the Doppler amplitude spectrum, we change the convolutional kernel structure of [5 × 5] at positions C1 and C3 to [5 × 1] and the pooling structure of [2 × 2] at positions P2 and P4 to [2 × 1] in the network model. Assuming the dimensionality of the input temporal data is 512, multiple feature levels are obtained by two groups of 1D convolutional network layers and 1D Max pooling layers in turn. Then, the Flatten network layer is used to flatten multiple feature levels to obtain a feature vector of dimensional size 8000. At the end of this network module, we utilize two fully connected network layers for feature compression and optimization to obtain a feature vector of dimension 256.

**Figure 5:**
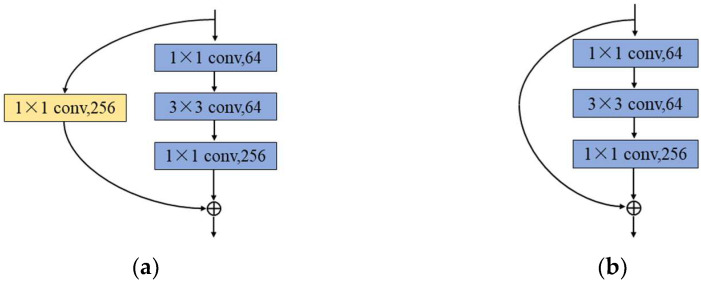
The network structure of the Conv Block and Identity Block in the Stage1 module. The 1 × 1 convolution kernel is a change in the dimension of the features. The structure of skip connection achieves the linear transfer of features. (**a**) The Conv Block network structure; (**b**) The Identity Block network structure.

For the time-Doppler spectrum image, we use the time-frequency feature extraction network in Figure 4b for feature extraction. The ResNet50 network model [31] is utilized as a backbone network to mine the spatial features in the time-Doppler spectrum image. Then, the features are compressed and optimized by a Global Average pooling layer and two fully connected network layers. Compared with other network models, the ResNet50 network model constructs a residual structure to solve the problem of inefficient feature learning and ineffective accuracy improvement due to the deepening of network layers. As shown in Figure 4b, the ResNet50 network model contains a total of five parts. The Stage0 mainly pre-processes the input image, it contains a convolutional layer and a pooling layer. From Stage1 to Stage4, each module contains a Conv Block (located in the first block) and multiple Identity Blocks. The network structure of the Conv Block and Identity Block in the Stage1 module is shown in Figure 5. The residual learning of features is achieved by linear transfer of the previous network layers to the later ones via the structure of skip connection. The Conv Block has different dimensions of input and output and its role is to change the dimensionality of the features. The Identity Block has the same dimension of input and output for deepening the number of network layers. Assuming that the input spectrum image size is 256 × 256 × 3, the features with dimension size 8 × 8 × 2048 are obtained by the ResNet50 network, in which 2048 is the number of feature levels and 8 × 8 is the dimension of each feature level. Then, we use the Global Max pooling layer for feature compression to obtain the feature vector with a dimension of 2048. Finally, we also use the two fully connected network layers to obtain a feature vector of length 256.

In the feature fusion stage, because the different modalities data are descriptions of the same observed sea surface, concatenating the feature vectors of different modalities data through the Cat network layer will result in redundant features. Therefore, we introduce a Self-Attention module [32] to learn the weight coefficients of different features for feature selection and reinforcement, causing the model to be more focused on discriminative features.

As shown in the Self-Attention module in Figure 3. First, the feature vectors are linearly transformed through the Embedding network layer to obtain three feature matrixes of equal size Q, K, and V, respectively. Then, Q and K are input into the MatMul network layer to perform matrix multiplication operation to obtain the similarity matrix of different features. In order to prevent the problem of vanishing gradients during the training of network parameters due to the oversized features, the scaling process is performed by the Scale network layer and Mask (opt.) network layer with a scale factor of $1/\sqrt{d_{k}}$, where $d_{k}$ is the feature dimension of the input self-attentive module. Finally, the similarity matrix is input into the SoftMax function for normalization to obtain the weight matrix of the features and the optimized feature vector is calculated by multiplying the weight matrix with V using the MatMul network layer. Equation (4) shows the calculation process of the Self-Attention mechanism.

(4) $$Attention(Q,K,V)=softmax(\frac{Q\times K^{T}}{\sqrt{d_{k}}})\times V$$

In the target detection stage, the extracted features and the SoftMax network layer are used for target and sea clutter decisions. The decision threshold is determined according to the output data of the training set and the desired false alarm probability. Meanwhile, we convert the binary hypothesis test problem for marine target detection into a binary classification problem for sea clutter and targets [18,19]. If the range cell contains the target, the echo signal contains the target echo, we can define the category of the echo signal and the different modalities data as the target, which is extracted from the echo signal. Otherwise, it is defined as sea clutter.

As shown in Figure 3, this paper uses two fully-connected network layers to compress the features from the Self-Attention module. The compressed features are entered into the SoftMax network layer and the predicted probability R, R∈[0,1] is calculated for the target. If *R* is greater than the decision threshold, the detected echo signal is considered to contain the target echo and is judged to be the target category. The SoftMax network layer is composed of a full-connected network layer and a SoftMax function. The output of the full-connected network layer is set to two probability values, one of which is the predicted probability (R) of the target category, and the other is the predicted probability of the sea clutter category. The sum of the two probabilities is 1. The SoftMax function is mainly used to normalize the data and it is used here to ensure that the sum of probabilities is 1. Suppose that $X_{0}=[x_{1},x_{2},\ldots ,x_{i},\ldots ,x_{N_{0}}]$ is a vector of length $N_{0}$ and $x_{i}$ are data in $X_{0}$, the output of $x_{i}$ through the SoftMax function is $x_{i0}$. The computation of $x_{i0}$ can be expressed as follows:

(5) $$x_{i0}=\frac{x_{i}}{\sum_{j=1}^{N_{0}}x_{j}}$$

For the decision threshold, in this paper, the prediction probability vector $R_{clutter}=[R_{1},R_{2},\ldots ,R_{N_{clutter}}]$ is obtained by the network model for the sea clutter samples in the training set and $N_{clutter}$ is the number of sea clutter samples. Under the false alarm probability $P_{fa}$ condition, the numbers of false alarm data i are calculated by Equation (6), respectively. The probability values in $R_{clutter}$ are sorted from big to small and then the i-th probability value is the decision threshold. The target samples in the testing set are classified by decision thresholds and the probability of detection ($P_{d}$) of the target is obtained by the statistics.

(6) $$i=P_{fa}\times N_{clutter}$$

### 3.2. Model Training

In the process of model training, the amplitude sequence, the Doppler amplitude spectrum, and the time-Doppler spectrum image of the echo signal are simultaneously input into the model, and the parameters of the network are optimized according to the loss function of the detection results and true categories. To reduce the training time of the proposed model, firstly, we construct the target detection network model based on single-modality data by adding a SoftMax network layer at the end of each feature extraction network module. Then, the target detection network model based on single-modality data is trained separately by using different modalities data to achieve the prediction of targets. Finally, we utilize the idea of transfer learning to load the weight parameters from the trained feature extraction network module into the feature extraction network module of the proposed model, which achieves the transfer of feature extraction capability between network models and the initialization of parameters in the proposed model. In the feature fusion stage of the model, to ensure that different modalities data have the same impact on the target detection network model, we ensure the length of the feature vector output by each feature extraction network module is 256.

The training process of the network model is carried out in the Tensorflow-gpu 2.1.0 environment. The computer configuration includes a CPU: Intel Xeon Silver 4210R, GPU: NVIDIA Quadro RTX 4000, and 64 GB of computer memory. In the network model, the batch size is defined as the number of samples for a train. Generally, a larger value of the batch size leads to a better optimization of the model parameters, but it also requires more memory resources. According to the memory of our computer, we define the batch size as 48. Besides, the learning rate (lr) indicates the scale of parameter update. If the lr is large, it will cause the model parameters not to converge. On the contrary, if the lr is small, the convergence of the parameters optimized will be slow. All things considered, we initialize the size of lr to 0.001 and then gradually become smaller with the number of iterations. Due to the initialization of the parameters by using the transfer learning method, the proposed model is in a relatively optimal state and only requires parameter fine-tuning to achieve fast convergence of the network model. Therefore, we set the iteration number of the network model to be only 100. The Adam optimizer [37] is used as the parameter optimization strategy.

(7) $$\theta_{j}=\theta_{j_{0}}-lr\frac{\partial }{\partial \theta_{j_{0}}}J(\theta )$$

where j are the number of trainable variables, $\theta_{j_{0}}$ are the original model parameters, $\theta_{j}$ are the optimized model parameters, and J(θ) is the loss function. The cross-entropy is used as the loss function,

(8) $$J(\theta )=-\sum_{k=1}^{K}\left[y^{\left(k\right)}(\theta )\log({\hat{y}}^{\left(k\right)})+(1-y^{\left(k\right)}(\theta ))\log(1-{\hat{y}}^{\left(k\right)})\right]$$

where K is the number of training samples, $y^{\left(k\right)}(\theta )$ is the detection probability of the model output, and ${\hat{y}}^{\left(k\right)}$ is the actual category label. We define the label of the target category as 1 and the label of the sea clutter category as 0. 

## 4. Experimental Results and Analysis

In this section, we utilize the data in Table 1 and Table 2 to construct the multimodal sample dataset for training and testing the network model. The experimental validation of the proposed model is performed under different sea states, different false alarm probabilities, and different marine floating target conditions. Additionally, it is compared with the feature-based detection methods [18,19] and the detection methods of single-modality data.

### 4.1. Dataset Construction

In this paper, by sliding sampling the long-time radar observation echo signal through Equation (9), we can obtain multiple echo signal sequences under short-time radar observation conditions. The multimodal data of the amplitude sequence, the Doppler amplitude spectrum, and the time-Doppler spectrum image are obtained by processing the radar echo signal sequence, i.e., the sample data of the network model.

(9) $$x_{j}(n)=x(d(j-1)+1:d(j-1)+D),j=1,2,\ldots ,N$$

where j is the number of samples, j∈N, d is the number of signal points in the sliding interval between adjacent samples, D is the length of the echo signal sequence, and D is calculated by multiplying the PRF with the observation time of the echo signal sequence.

For the sliding interval d, in this paper, in order to ensure the independent, identical distribution between neighboring samples, it is set by calculating the decorrelation time of the echo signal in each range cell. It is guaranteed that d is greater than the decorrelation time. Therefore, we set the sliding interval d for data #IPIX_01, #IPIX_02, #IPIX_03, #CSIR_03, #CSIR_04, and #CSIR_05 to 32 echo signal points and #CSIR_01 and #CSIR_02 to 64 echo signal points.

In addition, in order to ensure the reliability of the experimental results, it is necessary to ensure that the training set and the testing set are independent of each other. In this paper, the training set is built with the echo signal sequence of the first 2/3 observation times and the testing set is built with the echo signal sequence of the last 1/3 observation times. To avoid the class imbalance problem in the training set, the data ratio of sea clutter samples to target samples is about 1:1 using interval sampling of the sea clutter samples.

When the radar observation time is 0.512 s, we obtain the number of training and testing samples for different radar data in Table 3. For the IPIX radar dataset [33], this paper does not consider the electromagnetic scattering effect of the target on the adjacent cells and only uses the echo signal of the target cell and the sea clutter cell for sampling. Additionally, for the CSIR dataset [34], the echo signal of the guard cell is used as the sea clutter data for sampling. For example, for the data #IPIX_01, the guard cell is not considered. The sliding sampling using Equation (9) yields 32,652 training samples and 16,320 test samples, in which the data ratio between the target category and the sea clutter category is 1:11. The training data are equalized to obtain 2721 target samples and 2728 sea clutter samples. In addition, under the radar observation time of 0.512 s. For the IPIX radar data in Table 1, we obtain amplitude sequences and Doppler amplitude spectra with the data length of 512 and the time-Doppler spectrum image size of 256 × 256 × 3. For the CSIR data in Table 2, we obtain amplitude sequences and Doppler amplitude spectra with the data length of 5000 and the time-Doppler spectrum image size of 256 × 256 × 3 by reshaping the image.

According to the network parameters in Section 3.2, we train the proposed network model by using the training samples in Table 3. Then, the trained model is used to detect the training samples and the test samples, respectively. The detection probability of each sample as a target category is obtained from the SoftMax function. The detection probabilities $R_{clutter}$ of all training samples are sorted and calculated according to the required $P_{fa}$ to obtain the decision threshold. If the detection probability of the test sample is greater than the decision threshold, the test sample is the target category. The $P_{d}$ corresponding to the $P_{fa}$ is obtained by statistically calculating the results of the prediction category and the actual category for all testing samples.

### 4.2. Comparative Analysis with Single-Modality Data

In this section, the network model is trained by three groups of IPIX radar data with different sea states in Table 3. Using the test samples data, the detection results of the proposed method are compared with those of the single-modality data based on the amplitude sequence, the Doppler amplitude spectrum, and the time-Doppler spectrum image.

**Figure 6:**
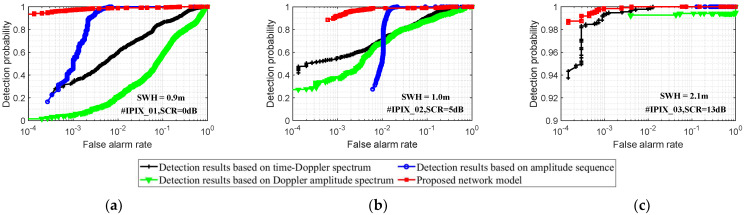
The proposed model is compared with the model based on single-modality data. The detection results are obtained from the test samples and the radar observation time is 0.512 s. (**a**) Lower SCR Sea state; (**b**) Medium SCR Sea state; and (**c**) Higher SCR Sea state.

Figure 6 shows the comparison of target detection results under different sea states. Where the radar observation target is a floating ball, the red curve is the detection result of the proposed model, and the blue curve, green curve, and black curve are the detection results based on the amplitude sequence, Doppler amplitude spectrum, and time-Doppler spectrum image, respectively. Compared with the detection methods based on single-modality data, the proposed model uses multimodal data information of the amplitude sequence, Doppler amplitude spectrum, and time-Doppler spectrum image so it can simultaneously characterize the target from different perspectives. Therefore, the proposed model has the best detection results under different SCR states and can maintain strong stability. In the lower SCR sea state of Figure 6a, when the false alarm probability is $10^{-3}$, the detection probability of the proposed model is 0.97, while the detection probability based on the amplitude sequence is only 0.45, the detection probability based on the Doppler amplitude spectrum is 0.05, and the detection probability based on the time-Doppler spectrum image is 0.33.

Due to the detection methods based on single-modality data, it can only describe the target and sea clutter from a single perspective while the ability to distinguish between the target and sea clutter is limited. Moreover, the detection performance of the model is also easily affected by the sea state environment. In Figure 6, it is obvious that the detection results based on single-modality data degrade to a large extent when the false alarm probability decreases. Especially in the lower SCR sea state conditions, the detection results of the different detection methods have more significant differences.

### 4.3. Performance Analysis under Different Sea States

**Figure 7:**
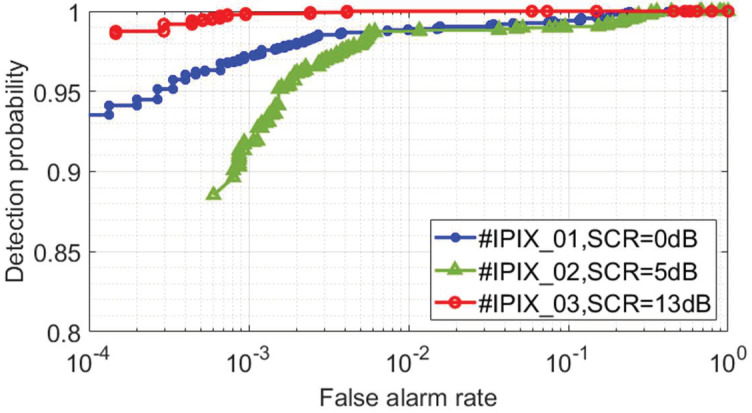
The detection probability of the proposed model under different SCR sea state data. The detection results are obtained from the testing samples and the radar observation time is 0.512 s.

In this section, we analyze the detection performance of the proposed model by using the IPIX radar data with three different SCR sea states in Table 3. Under the condition that the radar observation time is 0.512 s, we train the proposed model by using the training set sample data, and then the detection results in Figure 7 are obtained by detecting the trained model on the testing set samples. When the false alarm probability is $10^{-3}$, the detection results for the #IPIX_01, #IPIX_02, and #IPIX_03 data are 0.97, 0.92, and 1, respectively. The experimental results show that the proposed model can effectively detect the target in different sea states and has good detection results.

**Figure 8:**
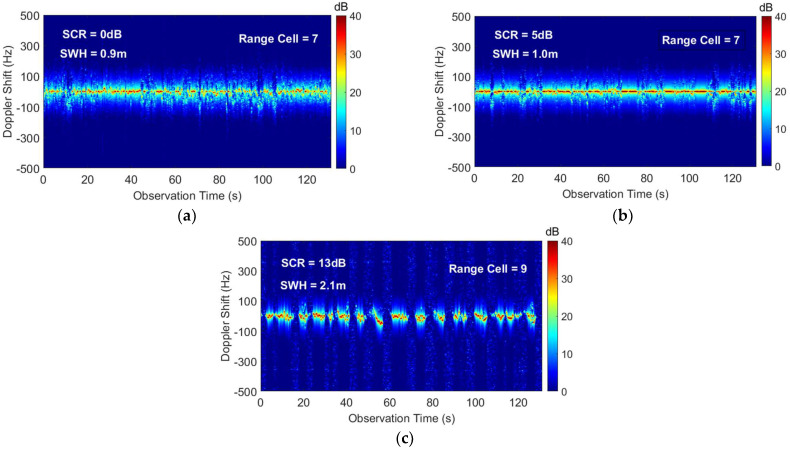
The time-Doppler spectrum image of the target cell for the IPIX radar data in Table 1. (**a**) The #IPIX_01 data; (**b**) The #IPIX_02 data; (**c**) The #IPIX_03 data.

Meanwhile, we can also conclude that the higher SCR corresponds to a larger detection probability. However, this relationship is not strictly monotonic because the sea state is an important factor affecting the performance [18]. For the data #IPIX_02 and #IPIX_03, the detection probability increases with the SCR. However, the data #IPIX_01 and #IPIX_02, we obtain the opposite result. We obtain the time-Doppler spectrum image of the IPIX radar data in Figure 8 by using Equation (3). It can be seen that the target always fluctuates around the zero-frequency and the motion states of the sea clutter and the target are similar. Compared with the #IPIX_02, the sea clutter in #IPIX_01 is far away from the zero-frequency due to the local gusts, and the distinction between target and sea clutter is enhanced. Additionally, it causes the sea clutter to have stronger echo energy and the target echo has relatively weaker energy. Therefore, the SCR of #IPIX_01 is smaller, but the experimentally obtained detection probability is higher. For #IPIX_03, the effect of the swell makes the floating target fluctuate to a greater extent. In Figure 8c, it can be seen that the separation of the target and the sea clutter is more obvious. Therefore, in Figure 7, the #IPIX_03 has the highest detection probability.

**Figure 9:**
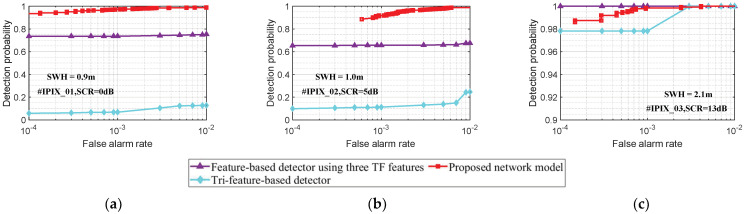
Comparison of different detection methods in different sea states. The detection results are obtained from the test samples and the radar observation time is 0.512 s. (**a**) Lower SCR sea state; (**b**) Medium SCR sea state; (**c**) Higher SCR sea state.

Furthermore, we use three sets of IPIX radar data to compare the performance of the proposed model and the feature-based detection method [18,19]. Under the condition that the radar observation time is 0.512 s, we utilize the convex hull algorithm to construct the Tri-feature-based detector [18] and the Feature-based detector using three TF features [19], respectively. The convex hull is trained by all the sea clutter samples and then the test samples are detected. Figure 9 shows the detection results of different detection methods, where the purple curve and cyan curve are the detection results of the Tri-feature-based detector and the Feature-based detector using three TF features, respectively, and the red curve is the detection result of the proposed model. For the data of the lower SCR sea state and medium SCR sea state, compared with feature-based detection methods, the proposed model can effectively avoid the interference of sea clutter and has the best detection results by obtaining complementary features from different modalities data. As shown in Figure 8a,b, the target and sea clutter are overlapped in the time-frequency domain, which causes the target detection probability to be low for the Feature-based detector using three TF features. In addition, the detection results of the same detector in Figure 9a are better than those in Figure 9b because of the better separation of the target and the sea clutter. For higher SCR sea state data, although the three detection methods have almost the same detection results, it can be concluded from Figure 8c that the target and sea clutter have a larger difference in TF distribution. Consequently, the Feature-based detector using three TF features has the optimal result. As the target is obscured by waves, some training sample data are affected, which causes the proposed model to show a small performance loss. Obviously, the proposed model is more suitable for detection tasks in various sea conditions by considering the stability of the target detection performance.

**Figure 10:**
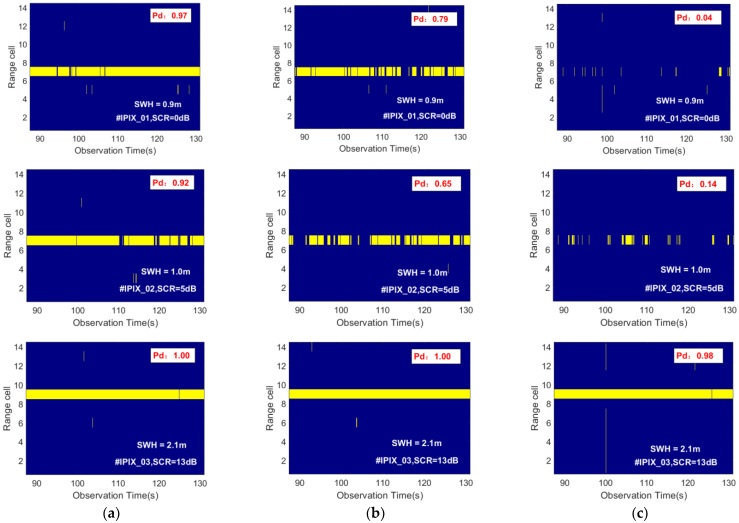
The detection results of different detection methods are visualized under different SCR sea state conditions. The detection result is obtained from the test samples. The radar observation target is the floating ball, the radar observation time is 0.512 s, and the false alarm probability is $10^{-3}$. The detection results of data #IPIX_01, #IPIX_02, and #IPIX_03 are shown from top to bottom by the row. The yellow marker is the detected target location. (**a**) Proposed detector; (**b**) Feature-based detector using three TF features [19]; (**c**) Tri-feature-based detector [18].

Figure 10 shows the detection results visualized for different detection methods on data #IPIX_01, #IPIX_02, and #IPIX_03 with a false alarm probability of $10^{-3}$. It can be seen that under the lower SCR sea state, the detection probability of the proposed model is 0.97, while the detection probabilities of the Tri-feature-based detector and the Feature-based detector using three TF features are 0.04 and 0.79, respectively. The influence of sea clutter on the target is different under different sea states. The characterization capability of the feature-based detector is limited and it is difficult to apply to the detection requirements of different sea states. Additionally, as the sea state changes, the detection results of the feature-based detector will fluctuate to a large extent. Comparing the detection results under different sea states, it can be concluded that the feature-based detection method showed great instability with the change of the sea state. In contrast, the proposed model has better detection results and good robustness under different sea states.

### 4.4. Performance Analysis of Different Marine Floating Targets

**Figure 11:**
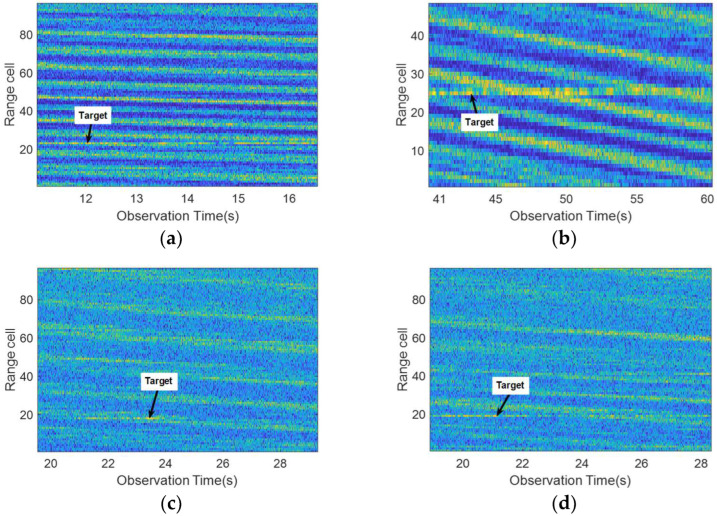
Radar echo intensity images of different marine floating targets. The images are obtained from the test samples, the radar observation targets are a floating fishing boat and a floating RIB from top to bottom by row, and the arrows point to the motion trajectory of the targets. (**a**) Data ID: #CSIR_01; (**b**) Data ID: #CSIR_02; (**c**) Data ID: #CSIR_03; and (**d**) Data ID: #CSIR_04.

In order to demonstrate the universality of the proposed detection model for the different marine floating targets detection, the sample data of #CSIR_01, #CSIR_02, #CSIR_03, and #CSIR_04 in Table 3 are selected for the training and testing of the network model in this paper. Figure 11 shows the radar echo intensity images of different marine floating targets, where the images show the echo data of the test samples and the arrows point to the motion trajectory of the targets. The radar observation targets for the data in the first and second rows in Figure 11 are a floating fishing boat and a floating RIB, respectively. The comparison shows that the sea state environment as well as the motion state of the target exhibit large differences for different data.

**Figure 12:**
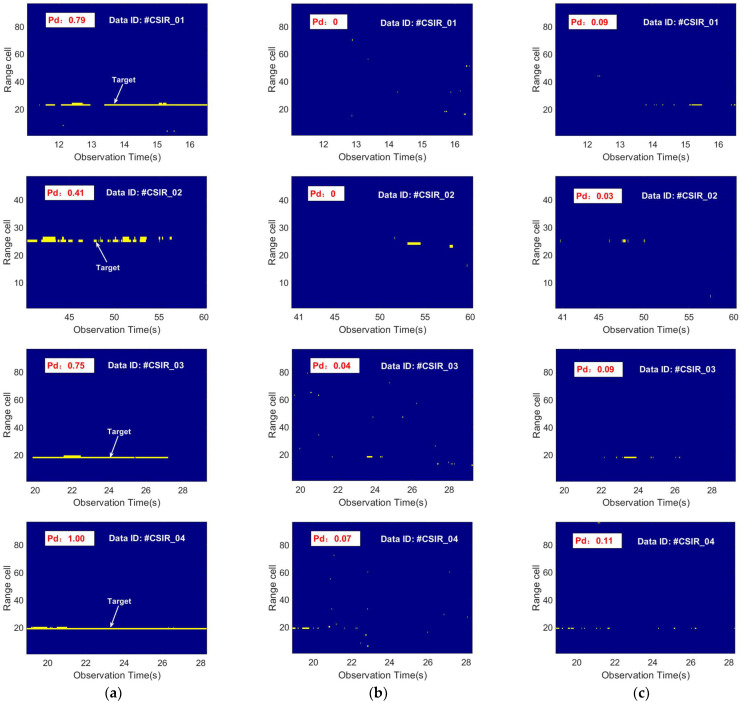
The detection results of different detection methods on different marine floating target data are visualized. The detection result is obtained from the test samples. The radar observation time is 0.512 s and the false alarm probability is $10^{-3}$. The radar observation targets of the data in the first and second rows are floating fishing boats and the data in the third and fourth rows are floating RIB. The arrow points to the correct target location for detection. The yellow marker is the detected target location. (**a**) Proposed detector; (**b**) Feature-based detector using three TF features [17]; and (**c**) Tri-feature-based detector [16].

Figure 12 shows the visualization of the detection results of different detection methods for the echo data in Figure 11, where the radar observation time is 0.512 s, the false alarm probability is $10^{-3}$, and the first-fourth rows show the detection results of data #CSIR_01, #CSIR_02, #CSIR_03, and #CSIR_04, respectively. It can be seen that on the data #CSIR_01, the detection probabilities of the proposed model, the Feature-based detector using three TF features, and the Tri-feature-based detector are 0.79, 0, and 0.09, respectively. The proposed model has the most optimal results. Similarly, for the data #CSIR_04, the detection probabilities of the three detection methods are 1.00, 0.07, and 0.11, respectively. By comparing different detection results, it can be concluded that the proposed model shows a strong universality and can be applied to detection tasks of different marine floating targets. The influence of wave motion makes it difficult for the feature-based detector to achieve high-performance detection of different marine floating targets. 

Table 4 shows the detection probabilities of different detection methods with different false alarm probabilities. Compared with the feature-based detector [18,19], the proposed model can greatly improve the robustness in different target data by extracting more discriminative features that produce stronger complementary effects. Under false alarm probabilities of $10^{-2}$, $10^{-3}$, and $10^{-4}$, the proposed model shows a higher detection probability. We can conclude that the feature-based detector is severely affected by sea surface variations due to the sea clutter is used as reference data to extract target features, which leads to difficulty in obtaining precise target decision thresholds. Moreover, the target detection performance of the feature-based detector decays to a large extent with the decrease in the false alarm probability and the detection results on different target data show a large instability.

In Table 5, the computational complexity of the proposed model is compared. At the stage of obtaining the sample data, the proposed model and the tri-feature-based detector using the TF features are more time-consuming because the time-Doppler spectrum image needs to be computed. In addition, the feature-based detection method requires the operation of extracting features from the spectrum image, so the tri-feature-based detector using the TF features has the longest time. In the test stage of the sample data, due to the computational complexity of the convex hull model increases with the number of training samples, the feature-based detection method is shorter on #CSIR_01 than on #CSIR_04. Besides, for the network model proposed, the forward propagation computation of the neural network consumes less time, hence the time for testing the samples is relatively shorter as well. The comparison of these two time-metrics indicates that the proposed model is more time-consuming. However, combined with the detection results in Table 4, the detection performance of the proposed model in this paper is more competitive. In the field of deep learning, the detection time can be reduced to some extent by parameter clipping. This is a challenging research direction that we will explore in the future.

**Figure 13:**
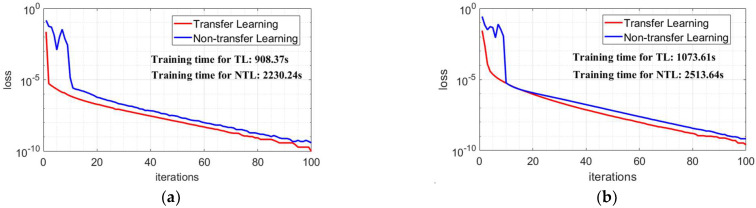
The loss of transfer learning and non-transfer learning in the model training process. The TL denotes the transfer learning method and the NTL denotes the non-transfer learning method. (**a**) #CSIR_01; (**b**) #CSIR_04.

To demonstrate the advantages of training the model by using the transfer learning in this paper. The transfer learning model is obtained by initializing the proposed model parameters based on the model parameters of the single-modality data. While the non-transfer learning model is obtained by without initializing the model parameters. In model training, we uniformly set the batch size of samples to 8, the number of iterations to 100, and the parameter optimizer to Adam. In Figure 13, the training losses and training times of the two training methods obtained by using different target data are shown. The training loss is obtained by J(θ). As the model parameters are initialized by using the transfer learning, a smaller model loss can be obtained with a shorter number of iterations and, also, the training time of the model is shorter. This provides the possibility for the application of deep learning methods to marine real-time target detection.

## 5. Conclusions

In this paper, a target detection network model based on multimodal data fusion of radar echoes is proposed. The multimodal data of amplitude sequences, Doppler amplitude spectra, and time-Doppler spectral images are obtained from the time domain, frequency domain, and time-frequency domain. According to the characteristics of different modalities data, the T-LeNet network module and the time-frequency feature extraction network module are constructed to extract different echo features. The Self-Attention mechanism is used to solve the feature redundancy between different modalities data. By using the measured IPIX radar dataset and CSIR dataset for detection experiments, the following conclusions can be obtained: (1) The multimodal data fusion of radar echoes by deep learning networks can obtain more complementary features, which can effectively avoid the interference of sea clutter on the target. Compared with the feature-based detection method and the detection model based on single-modality data, the proposed network model shows higher detection performance and stronger stability under the lower SCR sea state and medium SCR sea state. Moreover, the proposed model has better universality on different target data. The detection probability of the proposed model can achieve 0.97 when the false alarm probability is $10^{-3}$ under the sea state with low SCR. (2) Due to the diversity of marine floating targets, it is difficult for the feature-based detection method to obtain precise decision thresholds to discriminate the targets. Moreover, the target detection results fluctuate greatly with changes in the sea state, resulting in the inability to achieve high-performance detection. (3) The transfer learning method has a low training loss and can also effectively reduce the training time of the network model. It provides the possibility for the application of deep learning methods in marine real-time target monitoring. Moreover, the model is less time-consuming and has promising application prospects. In addition, the existence of rainfall changes the roughness of the sea surface as well as increases the attenuation of electromagnetic waves, which seriously interferes with the precise detection of targets [38,39]. Therefore, in future work, we will utilize more modality data to design the detection network model that adapted to complex sea environments such as rainfall and typhoons.

## Figures and Tables

**Table 1:** Description of the IPIX radar data used in this paper.

Data ID	File Name	Target Cell	Guard Cell	SWH (m)	WS (km/h)	Angle (Degree)	SCR (dB)	Target Type
#IPIX_01	19931109_191449_starea (HH polarization)	7	6,8	0.9	19	98	0	Floating ball
#IPIX_02	19931108_220902_starea (HH polarization)	7	6,8	1.0	9	97	5	
#IPIX_03	19931107_141630_starea (HH polarization)	9	8,10,11	2.1	9	6	13	

Note: Each datum includes 14 range cells and the length of time series at each cell is 2^17^. The SWH is significant wave height, the WS is wind speed, the SCR is signal-to-clutter ratio, and the Angle is the angle between the line of radar sight and wind direction.

**Table 2:** Description of the CSIR data used in this paper.

Data ID	File Name	Number of Range Cell	Observation Time(s)	SWH (m)	SCR (dB)	Target Type
#CSIR_01	TFA17_009	96	16.5530	2.26	7.3	Floating fishing boat
#CSIR_02	TFA17_011	48	60.3132	2.28	9.1	
#CSIR_03	TFC15_041	96	29.2998	3.04	3.3	Floating RIB
#CSIR_04	TFC15_044	96	28.3362	3.04	3.6	

**Table 3:** Number of training samples and testing samples for different radar data. Radar observation time for each sample is 0.512 s.

Data ID	Target Sample (Training Set)	Sea Clutter Sample (Training Set)	Target Sample (Testing Set)	Sea Clutter Sample (Testing Set)
#IPIX_01	2721	2728	1360	14,960
#IPIX_02	2721	2728	1360	14,960
#IPIX_03	2721	2728	1360	14,960
#CSIR_01	836	855	418	39,710
#CSIR_02	3115	3149	1557	73,179
#CSIR_03	3000	3040	1499	142,405
#CSIR_04	2899	2945	1449	137,655

**Table 4:** The detection probability of different detection methods with different false alarm probabilities. The radar observation time is 0.512 s. Bold highlights the comparison results.

Target Type	Data ID	False Alarm Rate			Detector
		10^−2^	10^−3^	10^−4^	
Floating fishing boat	#CSIR_01	**0.94**	**0.79**	**0.73**	**Proposed detector**
		0.23	0	0	Feature-based detector using three TF features [19]
		0.71	0.09	0.01	Tri-feature-based detector [18]
	#CSIR_02	**0.64**	**0.41**	**0.41**	**Proposed detector**
		0.03	0	0	Feature-based detector using three TF features [19]
		0.36	0.03	0	Tri-feature-based detector [18]
Floating RIB	#CSIR_03	**0.85**	**0.75**	**0.74**	**Proposed detector**
		0.60	0.04	0	Feature-based detector using three TF features [19]
		0.68	0.09	0.01	Tri-feature-based detector [18]
	#CSIR_04	**1.00**	**1.00**	**1.00**	**Proposed detector**
		0.79	0.07	0.01	Feature-based detector using three TF features [19]
		0.99	0.11	0.01	Tri-feature-based detector [18]

**Table 5:** Time consumption of different detection methods for the data of #CSIR_01 and #CSIR_04. The radar observation time is 0.512 s. The probability of false alarm is $10^{-3}$. Bold highlights the comparison results.

Data ID	Time-Consuming	The Detection Method		
		Proposed Model	Tri-Feature-Based Detector [18]	Feature-Based Detector Using Three TF Features [19]
#CSIR_01	Time ^1^	493.69	**0.49**	611.52
	Time ^2^	**14.52**	32.15	36.48
#CSIR_04	Time ^1^	617.05	**1.07**	759.81
	Time ^2^	**14.34**	65.22	90.51

Time ^1^ (ms): The time to obtain a sample data by data processing; Time ^2^ (ms): The time to test a sample data.

## Data Availability

Not applicable.

## References

[1] Ward K.D., Tough R.J.A., Watts S. (2013). Sea Clutter: Scattering, the K Distribution and Radar Performance.

[2] Chen X., Guan J., Huang Y., He Y. (2017). Radar low-observable target detection. Sci. Technol. Rev..

[3] Chen X.L., Guan J., Huang Y., Yu H.X., Liu N.B., Dong Y.L., He Y. (2017). Fine processing and application of radar low observable Moving Target. Sci. Technol. Rev..

[4] Chen X., Guan J., Liu N., Zhou W., He Y. (2014). Detection of a Low Observable Sea-Surface Target with Micromotion via the Radon-Linear Canonical Transform. IEEE Trans. Geosci. Res. Lett..

[5] Raynal A.M., Doerry A.W. (2010). Doppler characteristics of sea clutter.

[6] Toporkov J.V., Sletten M.A. (2007). Statistical Properties of Low-Grazing Range-Resolved Sea Surface Backscatter Generated Through Two-Dimensional Direct Numerical Simulations. IEEE Trans. Geosci. Remote Sens..

[7] Liu Y., Frasier S.J., McIntosh R.E. (1998). Measurement and classification of low-grazing-angle radar sea spikes. IEEE Trans. Antennas Propag..

[8] Roberts W. (2010). Adaptive Radar Signal Processing. Diss. Theses Gradworks.

[9] Yan Y., Wu G., Dong Y., Bai Y. (2022). Floating Small Target Detection in Sea Clutter Using Mean Spectral Radius. IEEE Geosci. Remote Sens. Lett..

[10] Wu X., Ding H., Liu N.-B., Guan J. (2022). A Method for Detecting Small Targets in Sea Surface Based on Singular Spectrum Analysis. IEEE Trans. Geosci. Remote Sens..

[11] Gini F., Greco M.V., Diani M., Verrazzani L. (2000). Performance analysis of two adaptive radar detectors against non-Gaussian real sea clutter data. IEEE Trans. Aerosp. Electron. Syst..

[12] Conte E., De Maio A. (2004). Mitigation Techniques for Non-Gaussian Sea Clutter. IEEE J. Ocean. Eng..

[13] Gini F., Farina A., Montanari M. (2002). Vector subspace detection in compound-Gaussian clutter. Part II: Performance analysis. IEEE Trans. Aerosp. Electron. Syst..

[14] Greco M., Stinco P., Gini F., Rangaswamy M. (2010). Impact of Sea Clutter Nonstationarity on Disturbance Covariance Matrix Estimation and CFAR Detector Performance. IEEE Trans. Aerosp. Electron. Syst..

[15] Lo T., Leung H., Litva J., Haykin S. (1993). Fractal characterisation of sea-scattered signals and detection of sea-surface targets. Proc. Inst. Elect. Eng..

[16] Li D., Shui P. (2014). Floating small target detection in sea clutter via normalised Hurst exponent. Electron. Lett..

[17] Hu J., Tung W.W., Gao J. (2006). Detection of low observable targets within sea clutter by structure function based multifractal analysis. IEEE Trans. Antennas Propag..

[18] Shui P., Li D., Xu S. (2014). Tri-feature-based detection of floating small targets in sea clutter. IEEE Trans. Aerosp. Electron. Syst..

[19] Shi S., Shui P. (2018). Sea-Surface Floating Small Target Detection by One-Class Classifier in Time-Frequency Feature Space. IEEE Trans. Geosci. Remote Sens..

[20] Guo Z.-X., Shui P.-L. (2020). Anomaly Based Sea-Surface Small Target Detection Using K-Nearest Neighbor Classification. IEEE Trans. Aerosp. Electron. Syst..

[21] Guo Z.-X., Shui P.-L., Bai X.-H. (2020). Small Target Detection in Sea Clutter Using All-Dimensional Hurst Exponents of Complex Time Sequence. Digit. Signal Process..

[22] Xu S., Bai X., Guo Z., Shui P. (2020). Status and prospects of feature-based detection methods for floating targets on the sea surface. J. Radars.

[23] Wang N., Wang Y., Er M.J. (2020). Review on Deep Learning Techniques for Marine Object Recognition: Architectures and Algorithms. Control Eng. Pract..

[24] Mu X. (2019). Radar moving target detection and classification based on time-frequency map deep learning. J. Terahertz Electron. Inf. Technol..

[25] Su N., Chen X., Guan J., Huang Y., Liu N. (2020). One-dimensional Sequence Signal Detection Method for Marine Target Based on Deep Learning. J. Signal Process..

[26] Su N., Chen X., Guan J., Huang Y. (2022). Maritime Target Detection Based on Radar Graph Data and Graph Convolutional Network. IEEE Trans. Geosci. Res. Lett..

[27] Pan M., Chen J., Wang S., Dong Z. A Novel Approach for Marine Small Target Detection Based on Deep Learning. Proceedings of the 2019 IEEE 4th International Conference on Signal and Image Processing (ICSIP).

[28] Chen X., Su N., Huang Y., Guan J. (2021). False-Alarm-Controllable Radar Detection for Marine Target based on Multi features Fusion via CNNs. IEEE Sens. J..

[29] Zhang C., Yang Z., He X., Deng L. (2020). Multimodal Intelligence: Representation Learning, Information Fusion, and Applications. IEEE J. Sel. Top. Signal Process..

[30] Baltrušaitis T., Ahuja C., Morency L.P. (2019). Multimodal Machine Learning: A Survey and Taxonomy. IEEE Trans. Pattern Anal. Mach. Intell..

[31] He K., Zhang X., Ren S., Sun J. Deep Residual Learning for Image Recognition. Proceedings of the IEEE Conference on Computer Vision and Pattern Recognition (CVPR).

[32] Vaswani A., Shazeer N., Parmar N., Uszkoreit J., Jones L., Gomez A.N., Łukasz K., Polosukhin I. Attention Is All You Need. Proceedings of the NIPS 2017—31st Conference on Neural Information Processing System (NIPS).

[33] The McMaster IPIX Radar Sea Clutter Database. http://soma.ece.mcmaster.ca/ipix/.

[34] De Wind H.J., Cilliers J.E., Herselman P.L. (2010). Dataware: Sea clutter and small boat radar reflectivity databases [best of the web]. IEEE Signal Process. Mag..

[35] Shui P., Bao Z., Su H. (2008). Nonparametric Detection of FM Signals Using Time-Frequency Ridge Energy. IEEE Trans. Signal Process..

[36] LeCun Y., Bottou L., Bengio Y., Haffner P. (1998). Gradient-based learning applied to document recognition. Proc. IEEE.

[37] Chen K., Ding H., Huo Q. Parallelizing Adam Optimizer with Blockwise Model-Update Filtering. Proceedings of the ICASSP 2020—2020 IEEE International Conference on Acoustics, Speech and Signal Processing (ICASSP).

[38] Lu Z., Sun L., Zhou Y. (2021). A Method for Rainfall Detection and Rainfall Intensity Level Retrieval from X-Band Marine Radar Images. Appl. Sci..

[39] Chen X., Huang W., Zhao C., Tian Y. (2020). Rain Detection From X-Band Marine Radar Images: A Support Vector Machine-Based Approach. IEEE Trans. Geosci. Remote Sens..

